# Benchmarking: A Tool for Veterinary Practices to Improve Prudent Use of Antibiotics in Cats and Dogs in Switzerland

**DOI:** 10.3390/antibiotics15010108

**Published:** 2026-01-22

**Authors:** Anaïs Léger, Heinzpeter Schwermer, Guy-Alain Schnidrig, Didier Wernli, Jacques Schrenzel, Dagmar Heim

**Affiliations:** 1 Federal Food Safety and Veterinary Office (FSVO), 3003 Bern, Switzerland; 2 Institute of Global Health, University of Geneva, 1211 Geneva, Switzerland; 3 Global Studies Institute, University of Geneva, 1211 Geneva, Switzerland; 4 Faculty of Medicine, University of Geneva, 1211 Geneva, Switzerland

**Keywords:** antibiotic, antimicrobial, companion animals, antibiotic use, benchmarking, veterinary practices, cats, dogs

## Abstract

**Background/Objectives**: Antibiotic use (ABU) in cats and dogs is a potential public health issue due to its direct contribution to the emergence of antibiotic resistance. In Switzerland, data on animal antibiotic treatments has been collected since 2020 via the Information System for ABU in Veterinary Medicine. This study focuses on the first implementation of a national benchmarking tool for ABU in cats and dogs in veterinary practices. **Methods**: The benchmarking tool is based on a practice-level indicator derived from the number of therapy days (pATI). Practices are compared separately for small animal practices and mixed practices, and for each animal species. The pATI is calculated based on the number of therapy days and is normalized by the number of consultations per species and per year. Practices were classified into four ABU categories based on their pATI: very high, high, acceptable, and no ABU. Thresholds for these categories are set according to Swiss legislation, using the 75th and 95th percentiles of the pATI values of all comparable practices. **Results**: By 2025, benchmarks were implemented in 686 veterinary facilities; a total of 667 (97.2%) received a pATI classification for ABU in dogs and 670 (97.7%) for ABU in cats. The median pATI was higher for cats than for dogs across all practice types. Similarly, the 75th and 95th percentile thresholds were also almost always twice as high for cats as for dogs across all practice types. **Conclusions**: To our knowledge, this is the first time a benchmarking tool for ABU has been implemented at a national level for cats and dogs. The benchmarking tool is expected to drive long-term changes in ABU practices.

## 1. Introduction

Responsible use of antibiotics (AB) in animals has been the focus of many interventions. So far, interventions have mainly targeted livestock animals, where the amount of AB used is considerably higher than in cats and dogs, and the risk of transmission of resistant bacteria to humans via food has been widely studied [1]. However, companion animals are also a source of the development of antibiotic resistance (ABR), as they are commonly treated with antibiotics for a range of infectious diseases [2]. The role of companion animals in the evolution and spread of ABR and its transmission to humans has become a topic of particular importance in ABR studies only recently [3,4,5,6,7]. The growing interest in this issue is linked to the changing attitudes towards companion animals in all societies. This relates particularly to their evolving relationship with humans, their increasing proximity, and the loosening of basic biosecurity rules [8,9], such as allowing animals to access bedrooms or lick their owners’ faces. It has been proven that the transmission of ABR genes between animals and humans in either direction is possible and can pose a risk to animal and public health [4,10].

Interventions and tools, such as international or national guidelines for prudent use and stewardship activities at clinics or teaching hospitals, have been implemented in diverse countries to guide veterinarians in the responsible use of AB [11,12,13,14,15]. Among available tools, benchmarking (BM) is recognized as a successful option. Benchmarking for antibiotic use (ABU) is “an intensive form of monitoring, designed so that actions can be taken to improve the health status of a population, and therefore frequently used in disease control campaigns” [16]. BM is intended to make it possible to determine the level of ABU, to compare ABU between veterinary practices, and to establish consequences (e.g., penalties or sanctions) in cases of excessive use. BM is recognized to have an indirect impact on ABU by increasing peer influence [17,18,19,20]. Benchmarking of ABU has already been implemented in several sectors, such as food-producing animals [21,22] and human health [23,24,25,26], providing a source of information for the implementation and the success of interventions. Benchmarking for companion animals is a less frequent practice and is only implemented in selected places, mainly on a voluntary basis, and never at the national level [27]. In South Korea, a study compared the ABU of 100 practices with the AB prescription data spanning 4 years [28]. In Germany, a study with data from 2018 to 2023 compared ABU in 165 practices that voluntarily provided their consultations and treatment information [29]. Another study recruited 2194 veterinary facilities among five veterinary clinic networks in the USA, Canada, and the UK between 2019 and 2021 to compare their ABU [30]. In the Netherlands, between 2012 and 2014, 100 veterinary practices were compared with each other [31,32]. However, these studies were not designed to be continuous or serve as a monitoring tool. In the United Kingdom, the Small Animal Veterinary Surveillance Network (SAVSNET) offers voluntary benchmarking for small animal practices providing their ABU data, with annual comparisons since 2017 [33]. It includes about 200 veterinary facilities, i.e., around 3% of UK registered facilities (200/6000, [34]). Thus, research on benchmarking for companion animals is missing, especially regarding implementation strategy and choice of BM indicators. Comparisons of ABU at practice level have been conducted on a small scale [28,29,30,35], but they do not report on limitations, hurdles, and successes that could arise when implemented at a national level. Also, these studies did not occur in an executive context where follow-up actions are directed at high users. By detailing our information system, measurement indicators, and BM tool, we aim to share our experience with our peers, helping them to tailor their interventions and learn from our successes and mistakes.

In addition, the European legislation requires ABU data to be collected yearly for cats and dogs from 2029 onwards [36]. This will raise interest and demand in most of the European Member States in the coming years. This study aims to investigate how to successfully implement a benchmarking tool for companion animal veterinary practices. We aim to provide evidence, feedback, and experience to guide adoption by other countries.

Several interventions and tools to improve ABU in companion animals have already been developed and implemented in Switzerland at the local or national level, namely treatment guidelines [37,38] and stewardship activities in selected places [39,40]. Furthermore, since 2020, the Federal Food Safety and Veterinary Office collects ABU data in cats and dogs with full national coverage (mandatory implementation for all veterinarians) with an information system for AB in veterinary medicine called IS ABV [41]. A first report analyzing ABU data was published in 2021, and reports have been available annually ever since [42]. These measures form part of the national health policy strategy against antibiotic resistance (StAR) and are enshrined in the Swiss legislation [43,44]. From 2024 onwards, a BM tool has been implemented at the practice level for ABU in companion animals. In 2024 and 2025, all Swiss veterinary practices received a summary report on their antibiotic use for cats and dogs. To our knowledge, this is the first time ever that such a BM tool has been implemented nationally.

This study will focus on the development, choice of indicator, and implementation of the BM tool for veterinary practices regarding use of AB in cats and dogs in Switzerland. We will focus on finding evidence that the Swiss approach provides relevant information that can be used to target ABU interventions in small animals’ veterinary practices identified as high users.

## 2. Results

### 2.1. Report for Benchmarking

**Figure 1:**
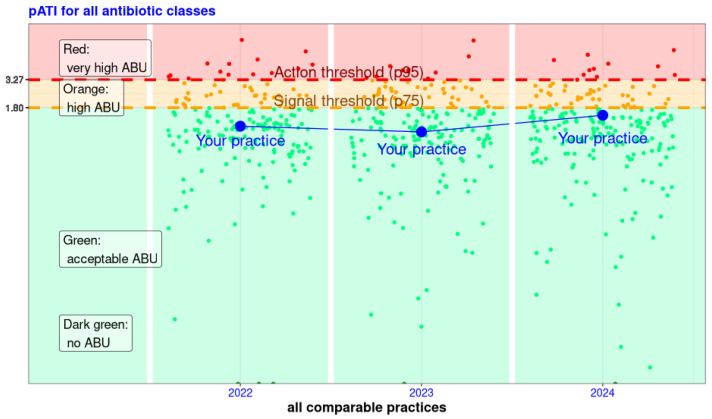
Logarithmic representation of the pATI (antibiotic treatment index at practice level) available in the reports for veterinarians, highlighting their result (in blue) and all other practices of the same practice type for the years 2022 to 2024—example of one of the six combinations of species and practice types. For a given year, each point represents the pATI of a practice. This allows the practice in the example (blue point and line) to see both its pATI in comparison with the pATI of all comparable practices and its evolution over the years. The pATI values are shown on the Y-axis, and the practices are shown on the X-axis in no particular order, per year. The colours are green for pATI values that show acceptable AB consumption. The orange area shows high consumption, and the red area shows very high consumption. These areas are divided by the threshold values: the signal value (75th percentile) and the action value (95th percentile). Thus, the practice in the example always has a pATI within acceptable consumption. In 2024, the pATI is higher than in previous years and only slightly below the signal value.

A report was sent to 989 Swiss practices that prescribed AB to cats and dogs from 2022 to 2024. Of these, 69.4% (686/989) received a report with a comparative analysis (pATI calculations, antibiotic treatment indicator at practice level) (Figure 1). Among the 686 practices, 202 (29.4%) were mixed practices, 414 (60.3%) were practices or clinics for companion animals with less than 4500 consultations per year, and 70 (10.2%) had over 4500 consultations per year. The 30.6% of practices that could not be benchmarked either had not registered their Nb*Consult* (number of consultations per species and per year) or registered less than 100 consultations per year. An anonymised report is available as an example in Supplementary Materials.

**Figure 2:**
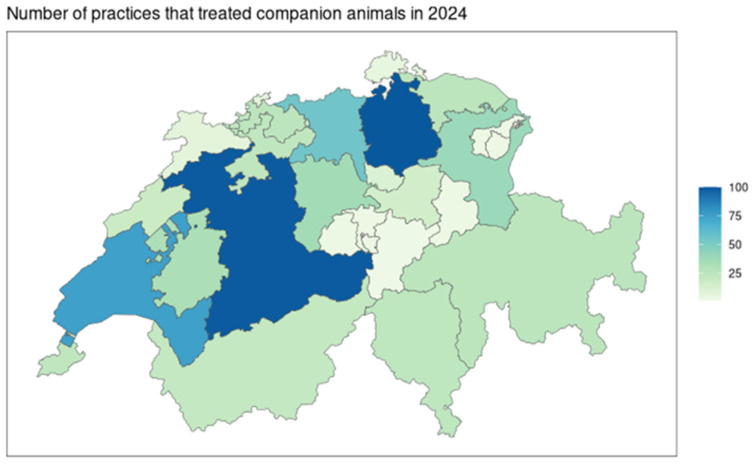
Representation per cantonal administrative regions of Switzerland of the number of practices that were included in the comparative analyses in 2024 (*N* = 686 practices).

The cantons of Bern, Zurich, Aarau, and Vaud gather 48.0% (329/686) of practices that declared consultations of cats and dogs (Figure 2). These four cantons represent 48.3% (224,189/464,447) of all antibiotic treatments in 2024 in Switzerland for cats and dogs.

### 2.2. Identifying High Users

**Figure 3:**
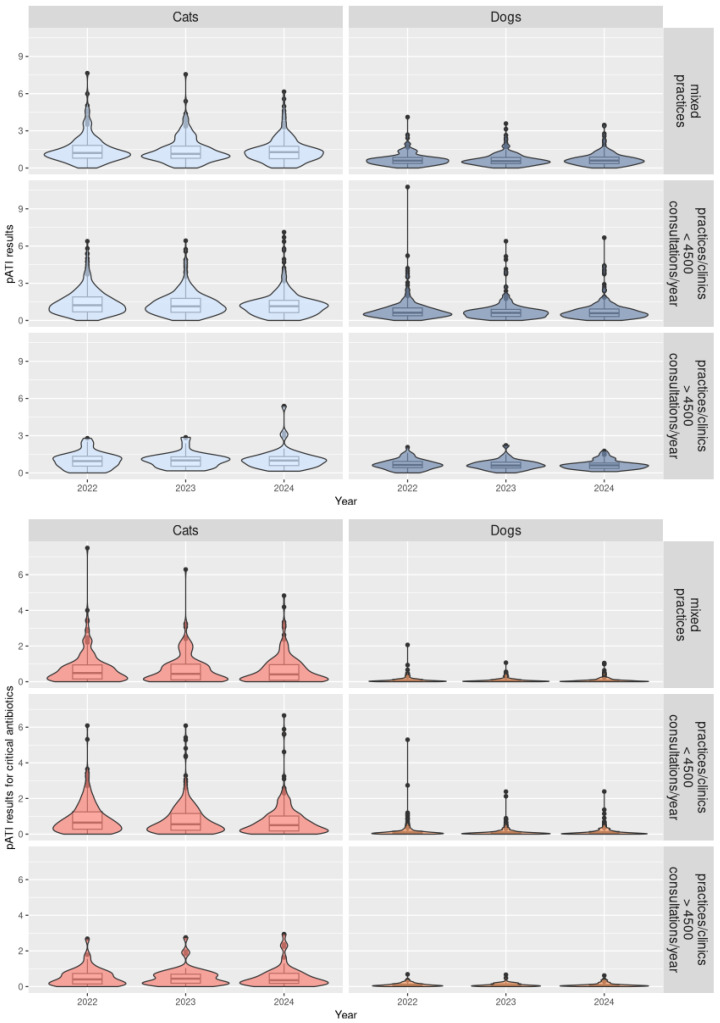
Distribution in violin plots of the pATI (antibiotic treatment indicator at practice level) results depending on the antibiotic classes (all antibiotics or critical antibiotics), species (cats or dogs), and type of practices for 2022 to 2024 (*N* = 686 practices).

Among the 686 concerned practices, 75.4% (517/686) were used to calculate signal and action thresholds. Results for the pATI calculations, thresholds, and categorisation are presented in Table 1, Figure 3, and Table A1 (Appendix A).

The results suggest differences in ABU patterns between cats and dogs, e.g., in AB used and the duration of therapy. Among the 686 practices, 667 (97.2%) received a pATI classification for ABU in dogs and 670 (97.7%) for ABU in cats. The median pATI for all AB and critical AB were higher for cats than for dogs for all types of practice (*p*-value < 10^−5^) (Table 1, Figure 3, and Table A2 in Appendix B). The signal and action thresholds were always higher for cats than for dogs for all types of practices (*p*-values < 0.006) and sometimes twice as high (Table A1 and Table A2 in Appendix A and Appendix B). In Figure 3, violin plots combine boxplots with usual values such as median and quartiles, as well as with coloured density curves. This helps display the numeric distribution of all the pATI values and better convey the distribution of practices for each species and practice type. For example, narrower coloured regions of the density plot indicate values that occur less frequently.

Signal and action thresholds for practices or clinics for companion animals with over 4500 consultations per year in both species were lower than for mixed practices and smaller practices or clinics, but this was not statistically significant with 2024 data (Table A1 and Table A2 in Appendix A and Appendix B).

Among the 686 practices, 47 (6.8%) practices were classified as very high users (above the action threshold) in 2024 with regard to their ABU for cats and/or dogs. Among these 47 practices, 17 practices had corrected their Nb*Consult*, explaining that more than 5% of practices were identified as very high users.

In the 32 practices classified as very high users regarding their ABU for dogs, 9 (28.1%) were also classified as very high users for critical AB and 15 (46.9%) as high users (above signal threshold). None of the 32 practices were classified as non-users of critical AB. According to their pATI in 2023, 46.9% (15/32) were already very high users and 31.3% (10/32) were high users.

In the 32 practices classified as very high users with regard to their ABU for cats, 21 (65.6%) were also classified as very high users for only critical AB, and 7 (21.8%) as high users. None of the 32 practices were classified as non-users of critical AB. According to their pATI in 2023, 62.5% (20/32) were already very high users and 25.0% (8/32) were high users.

### 2.3. Differences in AB Treatment Percentages Between Species and Practice Types

**Figure A1:**
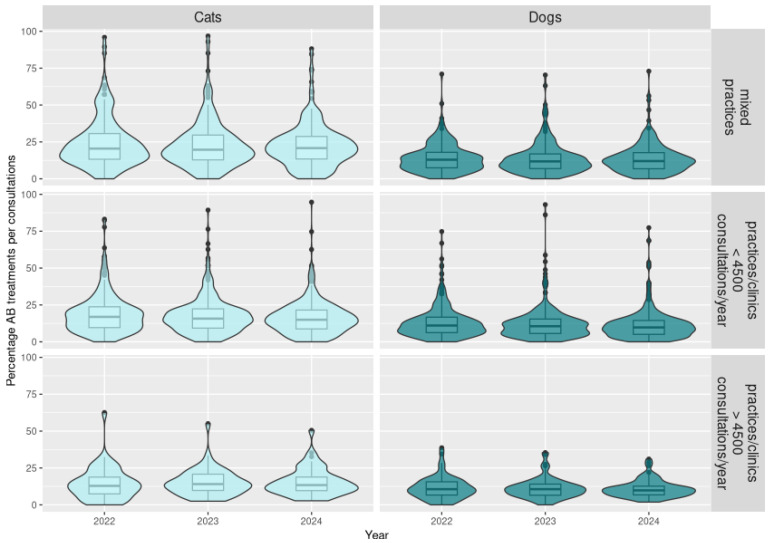

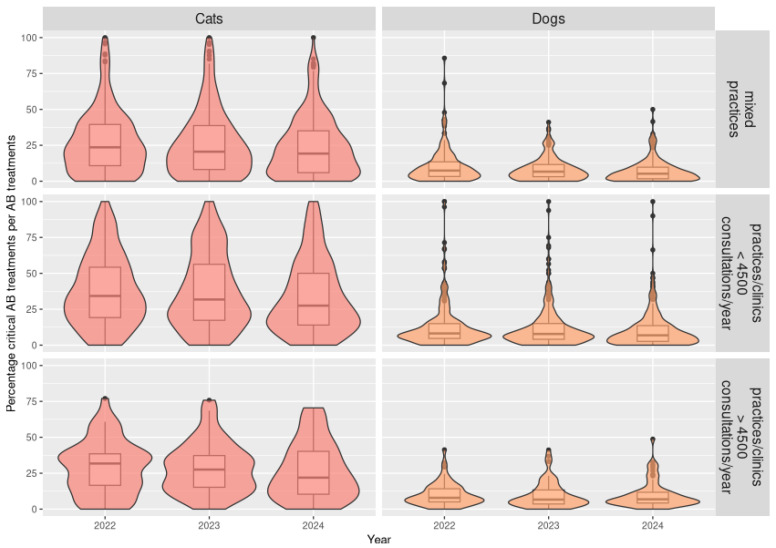
Distribution of the percentage of antibiotic treatments per consultation depending on the antibiotic class, species, and type of practice for 2022 to 2024 (*N* = 686 practices).

Consultations for cats in all types of practices resulted more frequently in the prescription of AB (between 13.6 and 21.1% in median) than for dogs (between 9.8 and 12.0% in median) (*p*-value < 10^−5^) (Table 1 and Table A2 and Figure A1 in Appendix B and Appendix C). The AB prescribed is more frequently a critical active substance for cats (median between 20.0 and 27.5%) than for dogs (median between 5.2 and 6.8%) (*p*-value < 10^−5^).

Regarding the number of consultations and the number of antibiotic treatments, mixed practices have a similar profile as practices or clinics with less than 4500 consultations per year (Table 1 and Table A2 and Figure A1 in Appendix B and Appendix C). However, when an animal visits a small animal practice or clinic, a consultation will less frequently involve a prescription of an antibiotic (median between 9.8% for dogs and under 15.0% for cats among all 414 practices) than when visiting a mixed practice (median 12.0% for dogs and 21.1% for cats among 202 practices) (*p*-value = 0.002). But in case of a prescription of AB, the antibiotic will more frequently contain a critical active substance in small animal practice or clinic (6.8% in median for dogs and over 22.3% for cats) than in a mixed practice (5.2% for dogs and 20.0% for cats) (*p*-value = 0.04).

## 3. Discussion

The choice of the indicator is at the core of benchmarking. Three groups of ABU indicators have been identified [45]: (1) count-based indicators [28,29,33], (2) weight-based indicators [28,30], and (3) dose-based indicators [28,29,30,32,35]. The Swiss indicator, pATI, is a measure of the number of days of active substance activity (i.e., therapy days). Antimicrobial use was quantified using pATI, which is a count-based ABU metric, rather than other weight- or dose-based metrics such as defined daily doses (DDD) or defined course doses (DCD) proposed by the European Medicines Agency [46]. DDD or DCD could be calculated using IS ABV prescription data [47]. However, for the national benchmarking systems for practices and livestock farms, the count-based (p)ATI offers the benefit of a very easy interpretation of the value on display. Comparisons between ABU measurement tools using count-based indicators, DDD, or DCD are challenging because of small differences in calculations, different data collection methodologies, different standard weights, and the definitions of the metrics used [47].

Results showed that practices exhibit different patterns of prescription depending on their type of practice. Mixed practices and small animal practices with less than 4500 consultations per year are similar in terms of number of consultations, prescriptions, and pATI thresholds. However, the pATI is higher in mixed practices than it is in all other practices and clinics, except for critical antibiotics. This could be explained by the profile of the practices: mixed practices are often used for first-level support and emergencies that require a quick reaction (e.g., abscesses or wounds); if the condition of the animal does not improve, or for follow-up, a consultation with a small animal practice or clinic might be needed. In this case, the small animal practice or clinic might need to administer critical antibiotics to the animal for second-line treatment. However, practices and clinics with more than 4500 consultations per year tend to have a smaller median pATI; this could be explained by better compliance with treatment guidelines, following training in AB therapies. Universities and clinics (e.g., numerous veterinarians, hospitalisation premises) were not excluded nor separated from analyses. However, their patients have a different profile from smaller practices, and they have the resources to implement antibiotic stewardship. In Switzerland, we separated small animal practices and clinics according to their number of consultations (i.e., their size) as it was not possible to differentiate clinics from practices in a systematic and meaningful way (i.e., no definition was available from the professional association). In foreign experiences, practices were classified according to workforce size [28] or the type of services they offered [30]; however, this type of information is not available in Switzerland. Our study also highlighted the significant differences in prescription habits between cats and dogs. This can be linked to the differences in veterinary medical products available for each species, to the different reasons for consultations, and to the management of animals by owners. For example, shelter treatments for abandoned or wild animals (i.e., mainly cats) are also included in the IS ABV database. These situations increase the use of AB treatments, especially for cats. It is, however, impossible to identify and exclude such treatments. In conclusion, supplementary analyses to better understand the profile of Swiss veterinary practices could be useful for further investigations of the need to classify practices and provide more meaningful categories.

The informative aspect of the report implies that no actions towards veterinary practices are taken until the thresholds are consolidated. However, we expect that the awareness of their own ABU compared to others will increase their voluntary prudent use by veterinarians. Although no measures are currently foreseen, the cantonal veterinarians responsible for the control of practices might use this data and request a more responsible ABU from high users.

In the following four months after the first release of the reports in 2024, we received feedback from 13 practitioners. The main themes of the contact were personal data modification requests, understanding of pATI calculation, and ways to improve their pATI results. Practitioners seemed genuinely interested and positive about the BM tool. They mainly tried to better understand the indicator for comparison as well as how their ABU could be improved. To increase acceptance and understanding of the report, we collaborated with members of the profession to provide a comprehensive description of the indicator, content of the report, and usefulness of this BM intervention. Veterinarians had access to newsletters, articles, and FAQs to facilitate the reading and ownership of the report. We assumed that practices would send feedback in case of discontentment, and that these interventions were therefore meaningful.

This first release of BM reports will hopefully start a long-term change in ABU prescription practices in Switzerland for companion animals. However, such an impact can be better assessed after several years of implementation. Interesting results and impacts of this benchmarking tool are not yet expected, as in other antibiotic stewardship experiences [35,48,49,50,51]. As no BM tool was ever implemented at the national level for companion animals, it is difficult to set targets and define predictions. To enhance meaningful changes, we provided complementary analyses as a first insight into veterinarians’ prescription habits. Thus, practices could start reflecting on their ABU and assessing their compliance with therapy guidelines. We intended to also provide a tool for antibiotic stewardship and improve not only the quantity of antibiotics used but also the quality of prescriptions (e.g., the right AB, at the right dosage, for the right indication). Assessing the impact of the BM should not only be based on the evolution of the total quantity of antibiotics or number of treatments but also on the adequacy of therapy guidelines. More analyses should be conducted in the future towards this last goal. That is why the BM report should evolve according to practice feedback and needs. ABU at the practice level is dependent on changes, such as scientific prescription recommendations (e.g., duration or dosage recommendations), “new” active substances or VMP, quantity of active substance per package, and price of VMP. Therefore, the development and evolution of the benchmarking indicator must be monitored over time to ensure effectiveness and stability. Also, further studies focusing on implementation strategies and implementation science might bring new perspectives on how to reflect on this first experience and bring a deeper understanding of obstacles and facilitators to improvement in ABU.

## 4. Materials and Methods

### 4.1. Data Collection

#### 4.1.1. IS ABV, the Information System for Antibiotic Use in Veterinary Medicine

Switzerland collects data on antibiotic treatments for all animal species via a web-based system called IS ABV (Information System for Antibiotic Use in Veterinary Medicine). All veterinarians have an obligation to send all their AB prescriptions and treatments, unless applied topically, since 2020. Thus, a coverage of 100% is expected from veterinary practices regarding their AB prescriptions. The following information must be sent for cats and dogs: the date of the prescription or application of the treatment, the targeted species, the weight of the animal, the indication of the treatment, the veterinary product used, the dosage, the duration and frequency of the treatment, and the quantity of the product sold. As IS ABV is currently structured, multiple entries cannot be combined into a single treatment or follow-up treatments. In this study, only data from 2022 to 2024 were used to conduct the analyses available in the BM tool. The collection process, content of the data collected, and the data quality management are described in a more detailed way in a separate paper [41]. Prescription data were extracted on 01.07.2025 from the IS ABV database.

The database allowed the identification of the antibiotic classes used, including “critical ABs”, i.e., macrolides, fluoroquinolones, and 3rd and 4th generation cephalosporines in Switzerland [52]. This classification is based on the World Health Organisation categorisation that identified “critically important antimicrobials” to be reserved for human medicine [53].

Data quality is a great concern, and several actions have been taken to improve the data collected [42]. Monthly feedback about potential errors is sent to practices to enhance veterinarians’ awareness of their own prescriptions. The current method for cleaning the database is described in a previous publication [54]. Veterinarians are legally responsible for the quality of the data they provide. As this benchmarking report is currently only informative, the hope is that it will incentivise practices to control their data and make corrections if necessary. For the current study, outliers, duplicates, and obvious errors were excluded from the dataset, as described in that paper [41].

#### 4.1.2. Number of Consultations

Since 2023, practices have been required to submit the total annual number of consultations per species, and 2022 was the first year for which this was mandatory. “Consultation” refers to any appointment at a veterinary practice or clinic that results in examination and/or treatment by a veterinarian, regardless of whether the animal is treated with antibiotics or other veterinary medicines. This number is used as the population at risk, i.e., cats or dogs that could receive an antibiotic treatment at the respective practice. Therefore, all analyses using the number of consultations were restricted to the years 2022 to 2024. The number of consultations was last extracted on 15 July 2025, to ensure the very last entries in IS ABV were included in the analyses.

Practices with less than 100 consultations per year were excluded from the benchmarking process, as they might not represent the “normal” use of AB (e.g., only emergencies and specialities not focused on companion animals).

Since 2023, practices are legally obliged to submit their Nb*Consult* by the end of February each year. Several reminders are sent via the IS ABV channels of communication (i.e., newsletter and monthly reports) to improve the quantity and quality of data collection.

#### 4.1.3. Types of Practices and Categorization

When registering in IS ABV, it is mandatory for practices to select a type of practice from a drop-down menu (e.g., practice for companion animals, clinic for companion animals, or mixed practice). Based on this information, this study categorized practices into three types: practice or clinic for companion animals with more than 4500 consultations per year, practice or clinic for companion animals with fewer than 4500 consultations per year, or mixed practice. BM was then applied in each type of practice independently.

We could not follow the commonly accepted classification of veterinary practices—i.e., general (primary) practice, emergency point-of-care (secondary), and referral and specialised (tertiary) clinic—as the number of clinics in Switzerland is not sufficient for an effective BM.

### 4.2. Indicator for Benchmarking

It is possible to compare practices by calculating one indicator for all veterinary practices, i.e., the number of ‘technical’ units of measurement normalized by the population at risk of treatment over a defined period [55]. The indicator should be adapted to the context and data collected.

#### 4.2.1. Antibiotic Treatment Indicator at Practice Level—pATI

The pATI is an AB treatment count-based indicator at the practice level derived from the number of days of active substance activity in all treated animals in relation to the population at risk in the practice. It was calculated following the formula below:

(1) $$pATI=\frac{\sum_{i=1}^{i=totVMP}\left(TD\left(i\right)\times NbAS\left(i\right)\times AT\left(i\right)\right)}{NbConsult}$$

The numerator is the sum of the therapy days [*TD*] for all veterinary medicinal antibiotic products (VMP) prescribed [*i*] (details are provided in Appendix D). The formula includes the number of active substances in the VMP [*NbAS*] multiplied by the number of therapy days. The formula also encompasses the number of animals treated [*AT*] with the prescription. Therapy days [*TD*] consider the days of treatment (i.e., when the treatment is applied) as well as the active substance effective time.

The denominator [Nb*Consult*] (i.e., population at risk) is the number of consultations by species provided by the practices on a yearly basis.

The pATI is calculated annually and independently for each species. No pATI was calculated for practices that did not provide their Nb*Consult* for any of the three years, or for those with fewer than 100 consultations per year. For practices that did not provide a Nb*Consult* for one or two years, we assumed the missing Nb*Consult* was the maximum of all registered years, and a pATI was calculated after correction.

The same process was implemented for critical antibiotics only: critical pATI.

#### 4.2.2. Thresholds for ABU Categorization

Two thresholds were defined to categorize practices according to their ABU: a signal threshold (75th percentile) and an action threshold (95th percentile). We adopted an approach based on percentiles for the identification of the higher users of AB among practices, as recommended [56]. These thresholds were calculated (1) based on the mean of 2022–2024 pATI values, excluding those based on corrected Nb*Consult*; (2) without the values of non-users (null pATI); (3) for each type of practice (i.e., practice for companion animals or mixed practices); and (4) for each animal species (cats and dogs).

All practices were then split into four categories according to their pATI results and within their practice type: very high ABU (above the action threshold), high ABU (between action and signal thresholds), acceptable ABU (below signal thresholds and not null), and no ABU (null pATI). Classification of practices was then displayed in a graphic, as presented in Figure 1.

The same process was implemented for critical antibiotics only, giving a second categorization of practices according to their use of critical AB.

#### 4.2.3. Quantile Regression for Comparing Medians, Action and Signal Thresholds

Quantile regression is suited for data with heterogeneous variance that is not normally distributed and for groups of different sizes. It was used to provide a robust comparison of two variables (pATI and percentage of AB treatments) between the animal species (cats and dogs) and between the practice types. We compared the 50% (median), 75%, and 95% percentiles of the 2024 data of these variables with a quantile regression with bootstrapping (Appendix B).

Statistical analyses were performed in R 4.4.2 [57] using the Fitting Linear Quantile Mixed Models Package (lqmm, version 1.5.8). The identification of the practice was used as a random effect, as most practices prescribed AB for both cats and dogs. All other parameters were set by default in the function lqmm. A *p*-value < 0.05 was considered a significant association. The function summary provided the results of the quantile regression with an *alpha*-value of 0.05.

### 4.3. Reports to Veterinarians

In August 2024 and 2025, veterinary practices received their BM report in a PDF document. The report was available in three Swiss official languages: German, French, and Italian. In addition to comparative data based on the BM indicator pATI, the report contained ABU analyses about the practice, with the aim of increasing knowledge about the quantity and quality of AB prescriptions.

The BM report had the same structure for all species: (1) comparative data and (2) a summary of ABU in the practice. The first part (1) covered the BM results and their presentation, as illustrated in Figure 1. Only the practice in question is identified in the graph; all other practices are displayed but anonymized. Practices with no pATI could see the repartition of practices, but their own practice was not on the graph. In the second part (2), all practices were able to compare their total number of treatments and total number of treatment days per animal with the Swiss median, as well as their percentage of AB treatments per Nb*Consult*, percentage of AB treatments with critical AB per AB treatment, and number of treatments per AB classes to the other practices with the same practice type. They also received their number of treatments per indication and per year, as well as the ten most prescribed VMP and the dosage used in the most recent year.

All data analyses and handling were carried out using R 4.4.2 [57]. Reports were built with R Markdown 1.12.

### 4.4. Exchanges with Selected Veterinarians or Professional Representatives

Throughout the process, a total of ten veterinarians and representatives of the profession were invited to comment and participate. Early in the process, we discussed the choice of indicator (pATI), with representatives of the veterinary profession and selected veterinarians interested in ABU management or already implementing stewardship in their practices. We also discussed the type of analysis that would be useful at the practice level and should be included in the second part of the report.

Later, we shared the final reports with three practices that were not involved in the previous discussions to obtain feedback on the quality, usefulness, and understandability of the report. We adapted the report according to their comments to improve readability.

## 5. Conclusions

To our knowledge, this is the first implementation of a national benchmark for veterinary practices for cats and dogs and, therefore, the first approach providing evidence on how to identify high AB users for further investigation and intervention. The novelty of our BM resides in (1) its national coverage and (2) its obligatory implementation. BM increases the understanding of Swiss prescription habits in cats and dogs and provides valuable information for improving ABU.

## Figures and Tables

**Table 1:** Overview of the main indicators for the summary of antibiotic use for all practices per species and type of practices based on 2024 data (*N* = 686 practices). Values for the critical antibiotics only are presented in brackets.

Data from 2024 (in Brackets the Value for Critical AB)	Number of Consultations Per Practice			Number of Animal Treatments * with AB Per Practice			Percentage of AB Treatments **	AB Treatment Indicator at Practice—pATI	Number of Practices
	Median	Mean	Max	Median	Mean	Max	Median	Median	
Dogs									
Mixed practice	1481	2130.2	19603	185.5 (8.0)	276.1 (22.2)	2202 (259)	12.0 (5.2)	0.60 (0.05)	202
Small animal practice or clinic < 4500 consultations/year	1612	1806.5	5068	150.0 (10.0)	203.7 (20.4)	2072 (412)	9.8 (6.8)	0.58 (0.06)	393
Small animal practice or clinic > 4500 consultations/year	5812	7584.6	45159	595.0 (45.5)	807.1 (84.7)	4164 (540)	9.8 (6.8)	0.60 (0.06)	72
Cats									
Mixed practice	1602	2054.3	13527	302.0 (68.0)	441.7 (106.7)	2218 (1289)	21.1 (20.0)	1.28 (0.41)	207
Small animal practice or clinic < 4500 consultations/year	1671	1908.7	6032	243.5 (64.0)	309.0 (96.6)	1583 (961)	15.0 (27.5)	1.14 (0.51)	414
Small animal practice or clinic > 4500 consultations/year	5545	6969.9	30920	819.0 (192.0)	1043.8 (256.5)	3420 (922)	13.6 (22.3)	1.00 (0.36)	49

* “Animal treatments” is the sum of the number of animals treated by species, practice, and year from all antibiotic prescriptions in the IS ABV database. ** Number of animals treated divided by the number of consultations. For critical ABs, we calculated the percentage of critical AB treatments among all AB treatments.

## Data Availability

The dataset presented in this article is not readily available because it contains the personal data of veterinary practices and veterinarians. However, requests to access the dataset and code can be directed to the appropriate channels of the FSVO. Requests to access the datasets should be directed to isabv@admin.blv.ch.

## Supplementary Materials

The following supporting information can be downloaded at https://www.mdpi.com/article/10.3390/antibiotics15010108/s1, Report S1: Antibiotic use data and comparative data.

## Abbreviations

The following abbreviations are used in this manuscript:

AB	Antibiotics
ABR	Antibiotic resistance
ABU	Antibiotic use
BM	Benchmarking
DCD	Defined course dose
DDD	Defined daily dose
IS ABV	Information System for ABU in Veterinary Medicine
Nb*Consult*	Number of consultations
pATI	Antibiotic treatment indicator at practice level
VMP	Veterinary medicinal antibiotic products

## Appendix A

**Table A1:** Signal and action thresholds for all and critical antibiotic classes, and detailed results of the pATI per species and type of practices based on 2024 data (*N* = 686 practices).

Data from 2024 (in Parathesis the Value for Critical AB)		Dogs			Cats		
		Mixed Practice	Small Animal Practice or Clinic < 4500 Consultations/Year	Small Animal Practice or Clinic > 4500 Consultations/Year	Mixed Practice	Small Animal Practice or Clinic < 4500 Consultations/Year	Small Animal Practice or Clinic > 4500 Consultations/Year
pATI values	Median	0.60 (0.05)	0.58 (0.06)	0.60 (0.06)	1.28 (0.41)	1.14 (0.51)	1.00 (0.36)
	Signal (p75)	0.87 (0.13)	0.93 (0.16)	0.88 (0.15)	1.80 (1.04)	1.80 (1.19)	1.27 (0.70)
	Action (p95)	1.57 (0.33)	1.76 (0.43)	1.47 (0.30)	3.27 (2.19)	3.10 (2.27)	2.00 (1.50)
Nb practices in each category of the benchmarking	Non-user	2 (34)	10 (55)	0 (2)	1 (22)	10 (26)	0 (2)
	Acceptable user	149 (128)	285 (245)	59 (56)	155 (137)	328 (313)	36 (34)
	Signal—high user	41 (30)	79 (78)	10 (9)	40 (38)	58 (61)	10 (9)
	Action—very high user	10 (10)	19 (15)	3 (5)	11 (10)	18 (14)	3 (4)
Percentage of practices that were used to calculate pATI-thresholds among all practices that received a pATI classification		75.7%—153/202	72.8%—296/393	73.6%—53/72	75.8%—157/207	75.1%—311/414	71.4%—35/49

## Appendix B

**Table A2:** Results of the quantile regression analyses (with bootstrapping) that compared medians, 75th, and 95th percentiles of pATI, pATI for critical AB, percentage of AB treatments, and critical AB treatments between practice types and animal species with a random effect from the identification of practices (*n* = 517 practices, data from year 2024 only). Significant results (*p*-value < 0.05) are highlighted in bold font and grey colour cells.

Variables Compared	Parameters of Quantile Regressions with Bootstrapping	Fixed Effects			AIC and Degrees of Freedom
		Practices > 4500 Consultations/Year	Mixed Practices	Dogs	
**pATI**	Γ = 0.50	Std Error: 0.05 *p*-value: 0.33	**Std Error: 0.52** ***p*-value: 0.006**	**Std Error: 0.02** ***p*-value: <0.00001**	1753 (df = 6)
	Γ = 0.75	Std Error: 0.08 *p*-value: 0.07	Std Error: 0.05 *p*-value: 0.09	**Std Error: 0.04** ***p*-value: <0.00001**	2010 (df = 6)
	Γ = 0.95	Std Error: 0.13 *p*-value: 0.79	Std Error: 0.16 *p*-value: 0.34	**Std Error: 0.09** ***p*-value: <0.00001**	2500 (df = 6)
**pATI for critical AB**	Γ = 0.50	Std Error: 0.01 *p*-value: 0.71	Std Error: 0.01 *p*-value: 0.69	**Std Error: 0.05** ***p*-value: <0.00001**	989.4 (df = 6)
	Γ = 0.75	Std Error: 0.03 *p*-value: 0.14	Std Error: 0.03 *p*-value: 0.39	**Std Error: 0.09** ***p*-value: <0.00001**	1532.7 (df = 6)
	Γ = 0.95	Std Error: 0.06 *p*-value: 0.92	Std Error: 0.07 *p*-value: 0.73	**Std Error: 0.30** ***p*-value: 0.006**	2177.3 (df = 6)
**pAAB**	Γ = 0.50	Std Error: 0.99 *p*-value: 0.10	**Std Error: 1.25** ***p*-value: 0.0002**	**Std Error: 0.38** ***p*-value: <0.00001**	7037 (df = 6)
	Γ = 0.75	Std Error: 1.77 *p*-value: 0.17	**Std Error: 1.53** ***p*-value: 0.005**	**Std Error: 0.43** ***p*-value: <0.00001**	7236 (df = 6)
	Γ = 0.95	Std Error: 2.15 *p*-value: 0.39	**Std Error: 2.57** ***p*-value: 0.004**	**Std Error: 0.82** ***p*-value: <0.00001**	7638 (df = 6)
**pAAB for critical AB**	Γ = 0.50	Std Error: 1.59 *p*-value: 0.56	**Std Error: 1.08** ***p*-value: 0.04**	**Std Error: 1.12** ***p*-value: <0.00001**	8537 (df = 6)
	Γ = 0.75	Std Error: 2.59 *p*-value: 0.83	Std Error: 2.50 *p*-value: 0.08	**Std Error: 1.43** ***p*-value: <0.00001**	8787 (df = 6)
	Γ = 0.95	Std Error: 3.58 *p*-value: 0.25	**Std Error: 5.31** ***p*-value: 0.046**	**Std Error: 4.68** ***p*-value: <0.00001**	7638 (df = 6)

## Appendix D

We used the following formula to calculate, per prescription (*k*) and veterinary medicinal antibiotic products (*p*), the therapy days (*TD*) of the prescribed treatment. Therapy days (*TD*) are the sum of the days of treatment (*TtmtD*, i.e., when the treatment is applied) and the active substance effective time (*AST*):

$$TD(k,p)=\left(TtmtD\left(k,p\right)-1\right)+AST$$

The active substance effective time is set per veterinary medicinal antimicrobial antibiotic products (VMP), species, and antibiotic. In the following table, we cannot provide the name of VMP, but we mentioned the name of the active substances.

**Table A3:** Values of days of active substance effective time used for the calculation of the antibiotic treatment indicator at the practice level (pATI) for antibiotic use in cats and dogs.

Antibiotic Class	Antibiotic	Days of Active Substance Activity
Cats		
Penicillin	Ampicillin	2
	Amoxicillin	2
	Benzathin-Penicillin	2
	Penicillin-Procain	2
Cephalosporin 3. generation	Cefovecin	14
All others	All others	1
Dogs		
Penicillin	Ampicillin	2
	Amoxicillin	2
	Benzathin-Penicillin	2
	Penicillin-Procain	2
Cephalosporin 3. generation	Cefovecin	10
All others	All others	1
