# Supplementary material for: Benchmarking: A Tool for Veterinary Practices to Improve Prudent Use of Antibiotics in Cats and Dogs in Switzerland
Source: Antibiotics (Basel). 2026 Jan 22;15(1):108. doi: 10.3390/antibiotics15010108 (PMC12837151; doi:10.3390/antibiotics15010108)
Supplement: Supplementary file 1 [file antibiotics-15-00108-s001.zip › antibiotics-4063491-supplementary.pdf]

Data on antibiotic use and comparative data

2022 - 2024

Cats and dogs

Practice information

This data corresponds to the information you entered in IS ABV.

**Practice**[a]	anonymised	**Type of practice**	Kleintier- und/oder Pferdeklinik
**Email**	anonymised	**UID-Nb**	anonymised
**Address**[a]	anonymised	**Supplement**	anonymised
**Canton**[a]	anonymised	**BUR-Nb**[a]	anonymised

[a] Source: BUR-Register

Evaluation status in 2024

Dogs : Your practice ATI is located in the orange category: high AB consumption.

Your practice ATI for critical AB is located in the green category: acceptable AB consumption.

Cats : Your practice TBI is located in the orange category: high AB consumption.

Your practice ATI for critical AB is located in the green category: acceptable AB consumption.

Schweizerische Eidgenossenschaft
Confédération suisse
Confederazione Svizzera
Confederaziun svizra

IS ABV
Informationssystem Antibiotika in der Veterinärmedizin

General

This report contains information on the use of antibiotics in cats and dogs at your practice during the specified period per calendar year. It consists of an overview and detailed evaluations per animal species and contains comparisons with data from other practices. All analyses are for your information only. The analyses refer to the years 2022 to 2024.

Prescriptions received that have been identified as invalid (unlikely prescription, see Reading aid), are excluded from the analyses in this report. See the following table: data delivered to the IS ABV and excluded data for 2024. A specific report with information on invalid prescriptions from your practice is available in the IS ABV web app.

Year	Species	Type of practice for comparison	Number prescr. with AB[a]	Number prescr. with crit. AB[b]	Number invalid prescr.
**2024**	Dogs	Practice for companion animals < 4500 consultations/year	359	63	0
**2024**	Cats	Practice for companion animals < 4500 consultations/year	1273	357	0

[a] AB = antibiotic

[b] krit. AB = critical AB, fluorchinolones, cephalosporines 3rd/4th generation, macrolides

Questions and comments regarding the analyses: isabv@blv.admin.ch

Report - as of 15.07.2025

Prescriptions - as of 14.07.2025

Consultations - as of 15.07.2025

Dogs – Comparative data

Total number of treatment days based on the number of consultations (pATI)

The antibiotic treatment indicator at practice level (pATI) is a key figure used to compare the intensity of antibiotic treatment in practices that treat companion animals. To do this, the pATI of all practices of a comparable type are compared. The pATI for pets corresponds to the total number of treatment days based on the number of consultations in the practice and thus reflects the intensity of treatment of animals with antibiotics. Click here for further details.

Year	Number AT[a] with AB	pATI numerator for all AB[b]	Number AT with crit. AB	pATI numerator for crit. AB	Number consultations	Evaluation status[b]
**2022**	785	4241	341	1953	1700	Complete evaluation possible
**2023**	624	3268	197	1026	1596	Complete evaluation possible
**2024**	359	1673	63	267	1700	Data incomplete, evaluation possible after correction

[a] AT = animal treatment, AB treatment of one animal

[b] Further details on the calculation and evaluation status can be found at the end of the document.

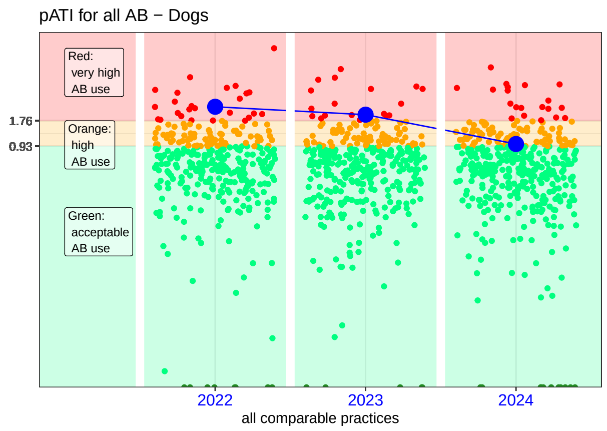

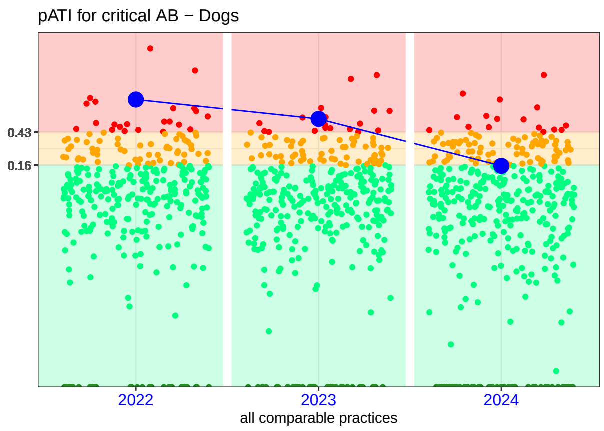

AB class	Year	pATI	Category for AB use	Practices with the same or lower pATI in %	Number of comparable practices
alle AB	2022	2.49	Red: very high AB consumption	96.8 %	393
alle AB	2023	2.05	Red: very high AB consumption	97 %	393
alle AB	2024	0.98	Orange: high AB consumption	77.9 %	393
kritische AB	2022	1.15	Red: very high AB consumption	99.1 %	393
kritische AB	2023	0.64	Red: very high AB consumption	98.1 %	393
kritische AB	2024	0.16	Green: acceptable AB consumption	76.3 %	393

Additional information: Total number of antibiotic treatments divided by the number of consultations

**Dogs**	**2022 - Your Praxis**	**2022 - Median CH**	**2023 - Your Praxis**	**2023 - Median CH**	**2024 - Your Praxis**	**2024 - Median CH**
Proportion of AB treatments/consultations (%)	46.2	11.1	39.1	10.6	21.1	9.8
Proportion of crit. AB treatments/ AB treatments (%)	43.4	8.2	31.6	7.8	17.5	6.9

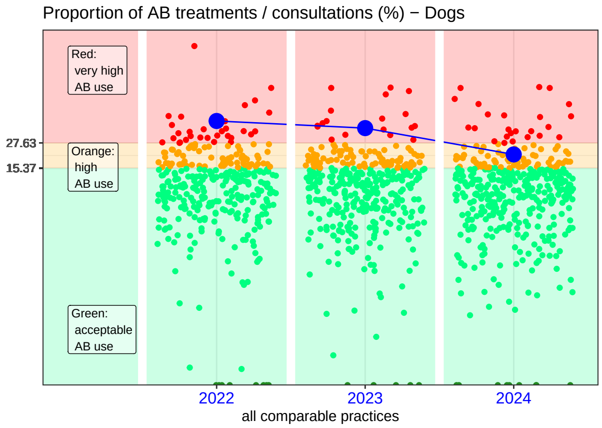

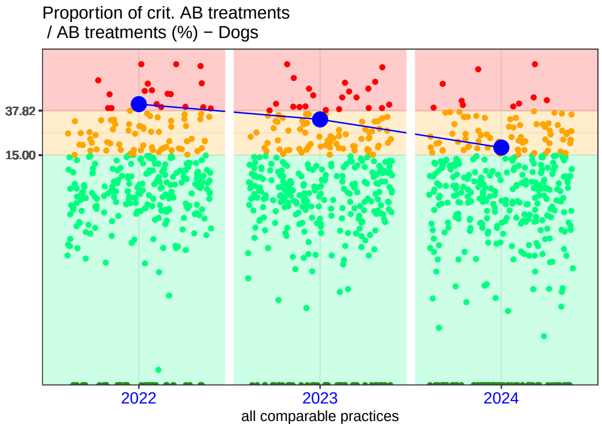

Dogs – Summary of your antibiotic prescriptions for the years 2022 to 2024

A reading aid for this data can be found at the end of the report (e.g. definitions, details on the calculations). Please note the different scales when comparing the graphs.

Animal treatments (TB)

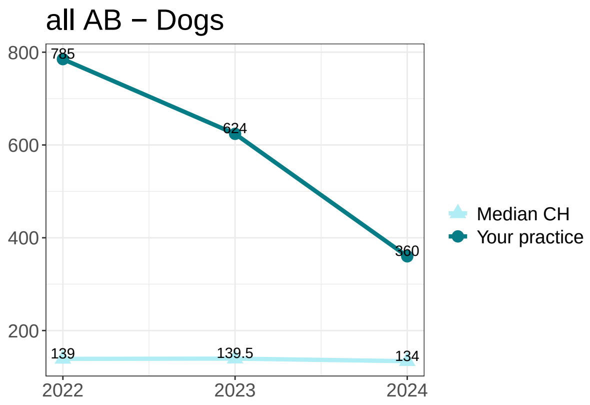

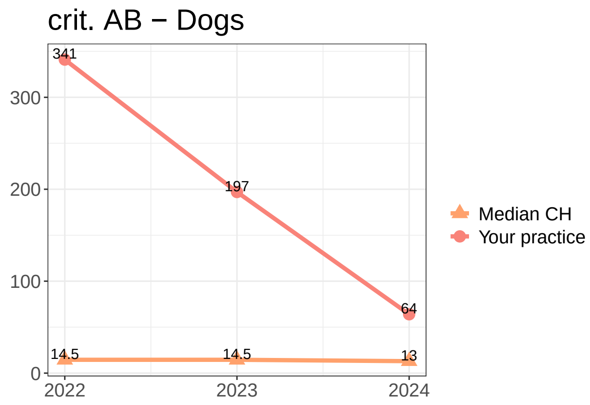

Number of therapy days per animal treated

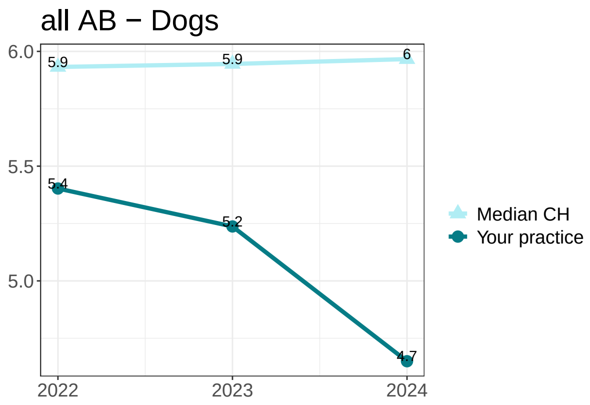

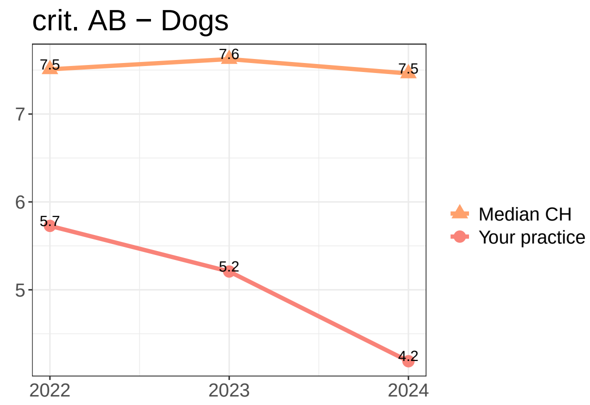

Number of animal treatments per active substance class

Your practice is represented by the blue dot (●); if it does not appear, you have not prescribed any antibiotics of the corresponding class. The other dots represent all practices of a comparable type with more than 100 consultations per year.

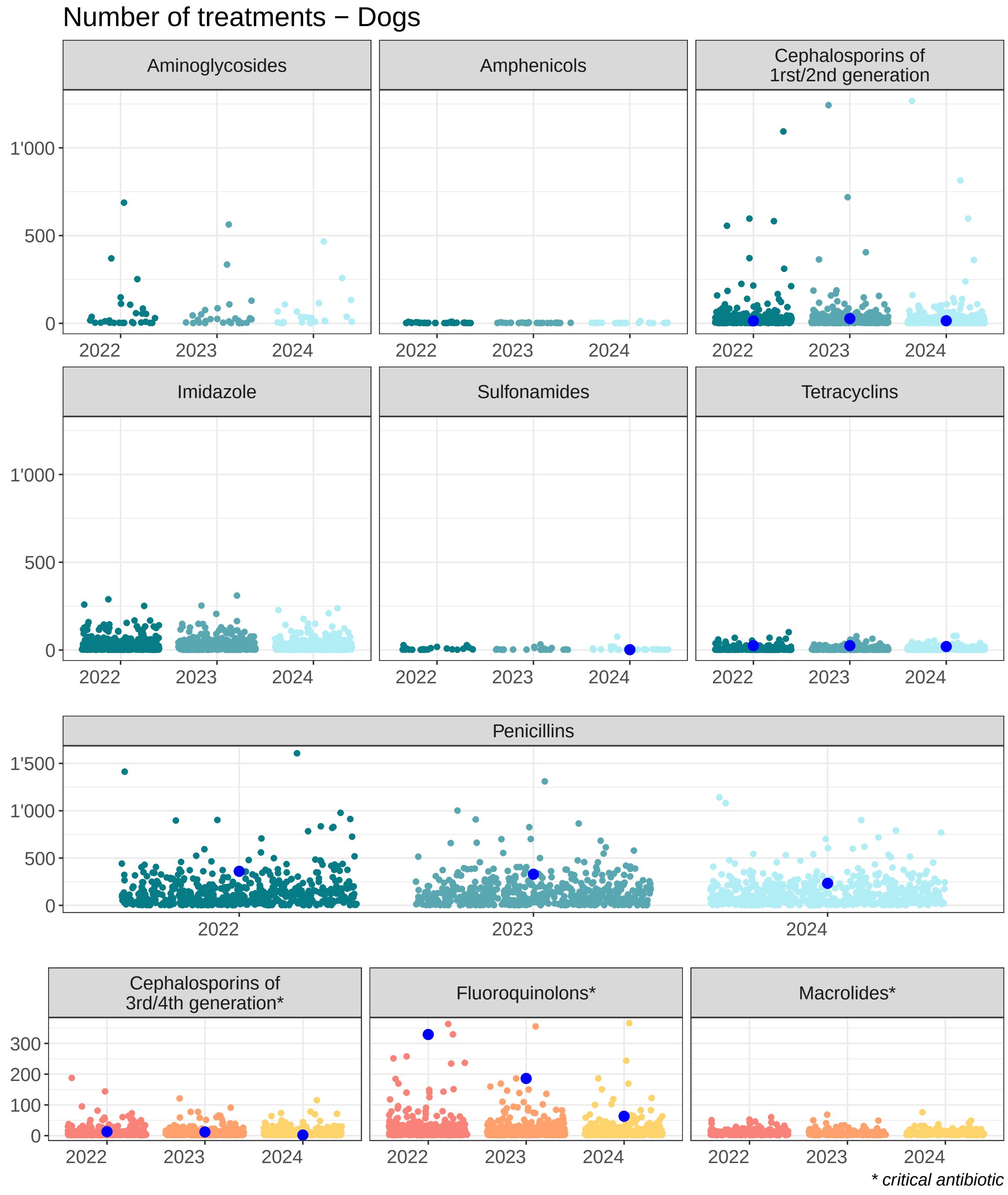

Animal treatments by indication

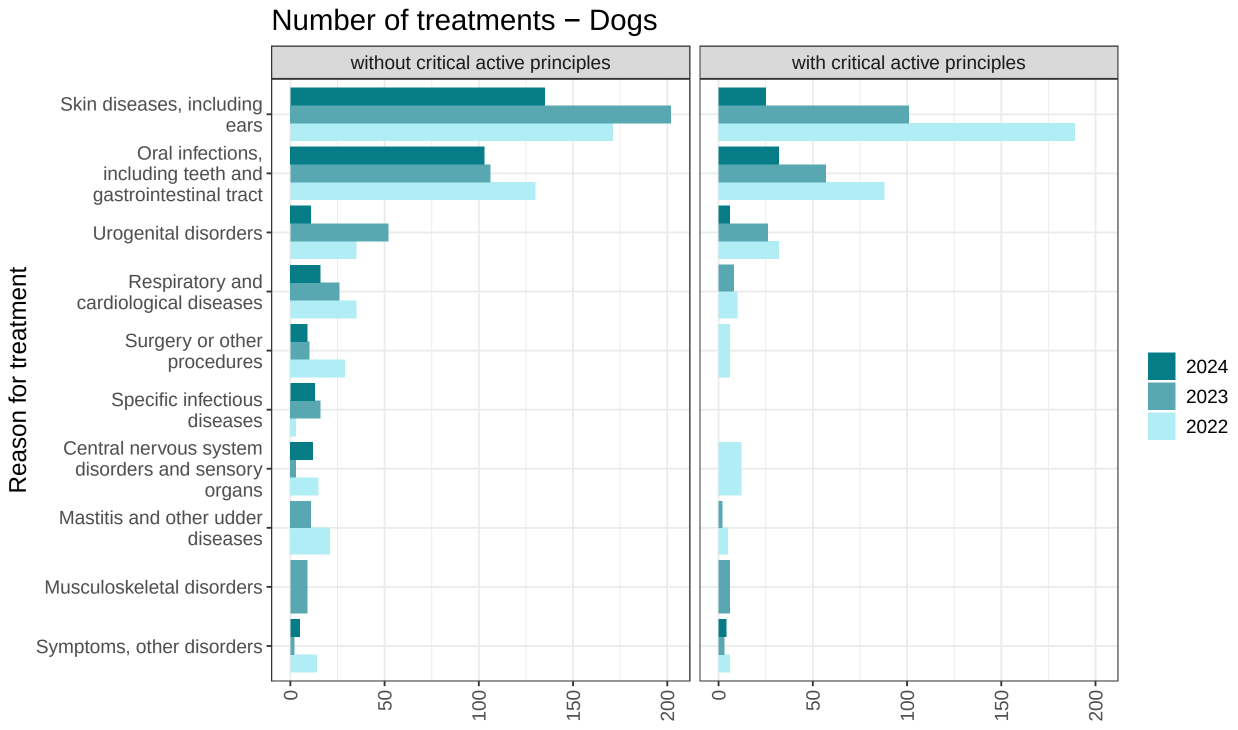

The 10 most frequently used preparations

This chart shows the 10 most frequently used preparations with which you treated the most animals in the last year.

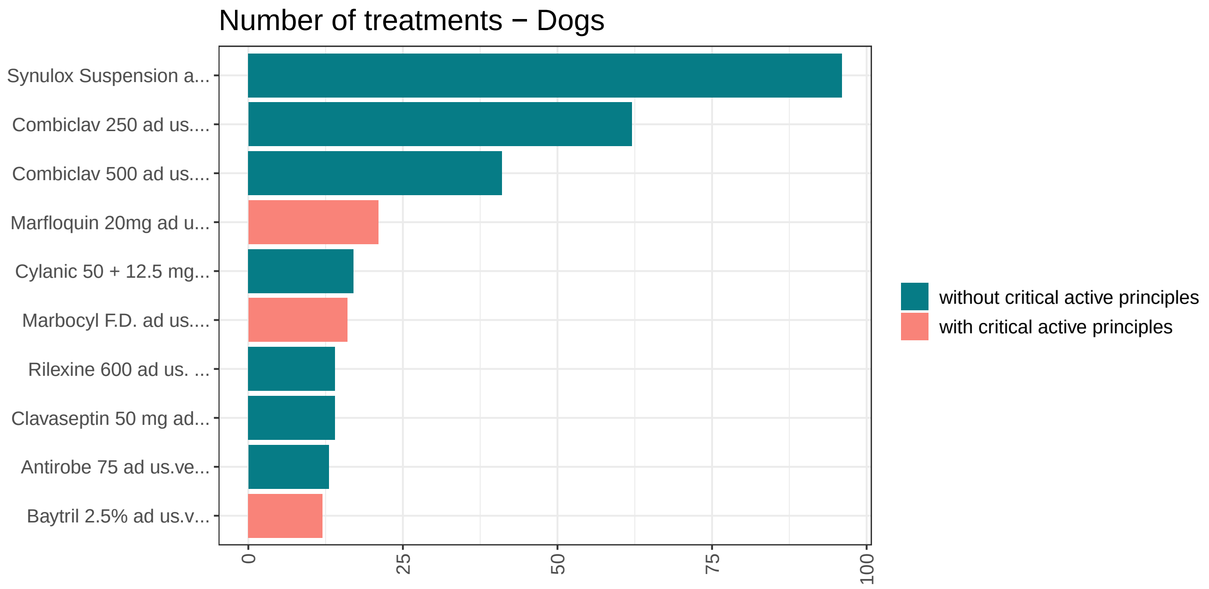

This graph shows all the dosages you prescribed for the preparations listed in the first graph for the year 2024 (amount of active ingredient administered per day and per kg of animal weight).

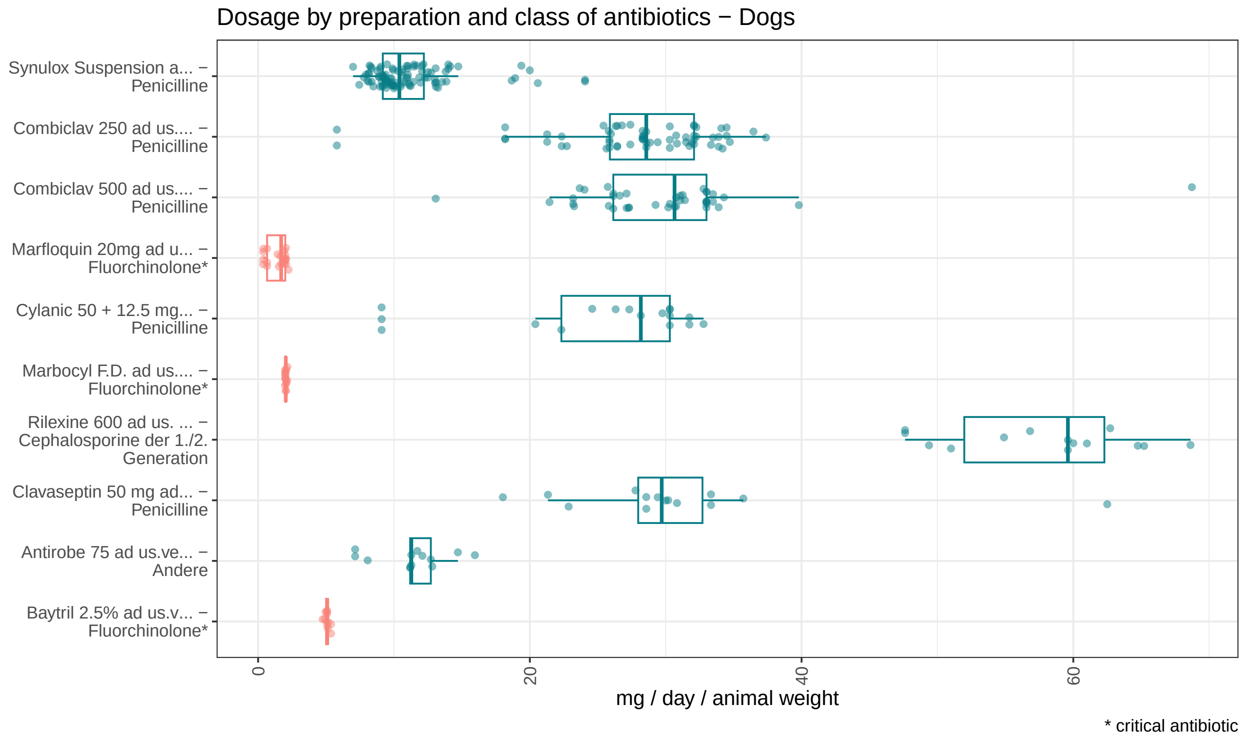

Cats – Comparative data

Total number of treatment days based on the number of consultations (pATI)

The antibiotic treatment indicator at practice level (pATI) is a key figure used to compare the intensity of antibiotic treatment in practices that treat companion animals. To do this, the pATI of all practices of a comparable type are compared. The pATI for pets corresponds to the total number of treatment days based on the number of consultations in the practice and thus reflects the intensity of treatment of animals with antibiotics. Click here for further details.

Year	Number AT[a] with AB	pATI numerator for all AB[b]	Number AT with crit. AB	pATI numerator for crit. AB	Number consultations	Evaluation status[b]
**2022**	2139	14529	1023	8091	3725	Complete evaluation possible
**2023**	1679	12346	656	6174	3505	Complete evaluation possible
**2024**	1273	8381	357	3470	3725	Data incomplete, evaluation possible after correction

[a] AT = animal treatment, AB treatment of one animal

[b] Further details on the calculation and evaluation status can be found at the end of the document.

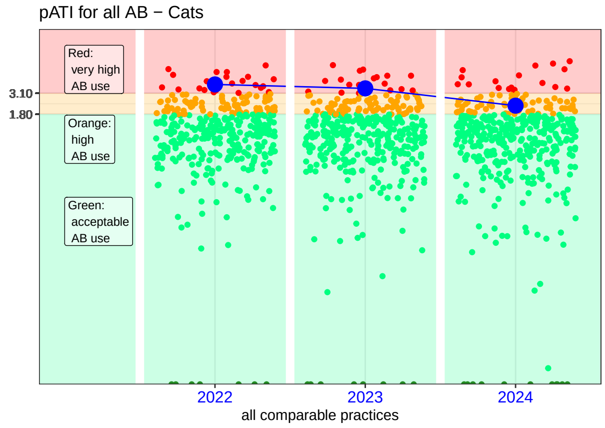

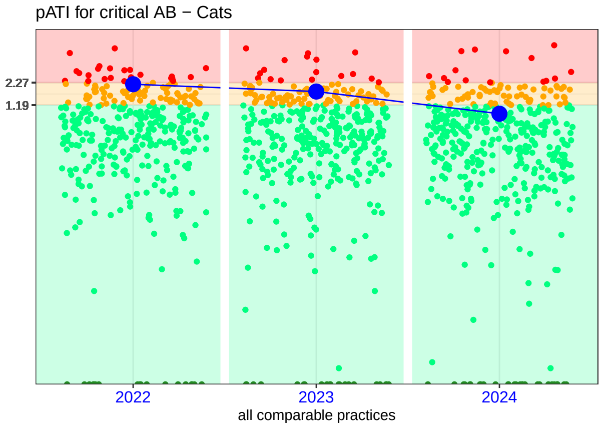

AB class	Year	pATI	Category for AB use	Practices with the same or lower pATI in %	Number of comparable practices
alle AB	2022	3.90	Red: very high AB consumption	97 %	414
alle AB	2023	3.52	Red: very high AB consumption	96.9 %	414
alle AB	2024	2.25	Orange: high AB consumption	88.6 %	414
kritische AB	2022	2.17	Orange: high AB consumption	92 %	414
kritische AB	2023	1.76	Orange: high AB consumption	90.4 %	414
kritische AB	2024	0.93	Green: acceptable AB consumption	72.7 %	414

Additional information: Total number of antibiotic treatments divided by the number of consultations

Cats	2022 - Your Praxis	2022 - Median CH	2023 - Your Praxis	2023 - Median CH	2024 - Your Praxis	2024 - Median CH
Proportion of AB treatments/consultations (%)	57.4	16.9	47.9	15.8	34.2	15.0
Proportion of crit. AB treatments/ AB treatments (%)	47.8	34.2	39.1	31.8	28.0	27.5

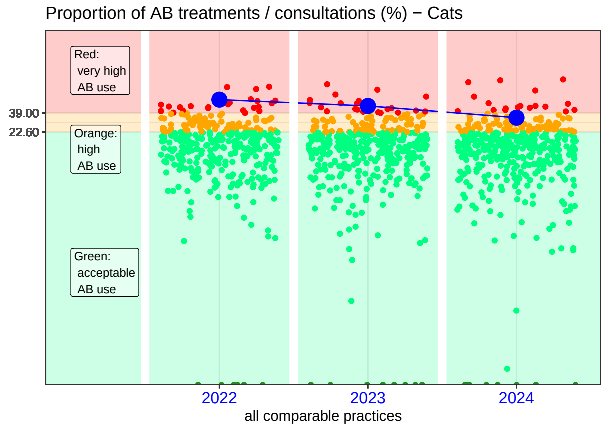

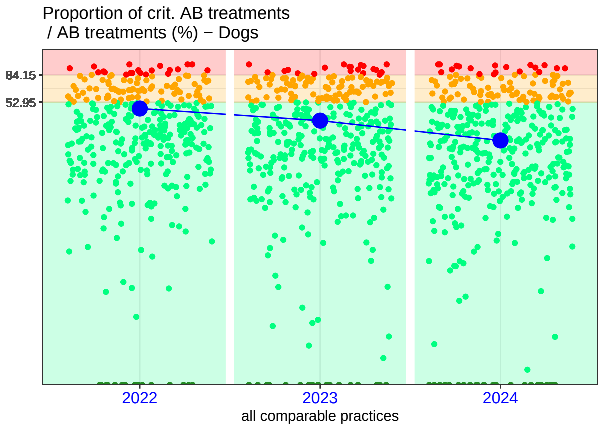

Cats – Summary of your antibiotic prescriptions for the years 2022 to 2024

A reading aid for this data can be found at the end of the report (e.g. definitions, details on the calculations). Please note the different scales when comparing the graphs.

Animal treatments (TB)

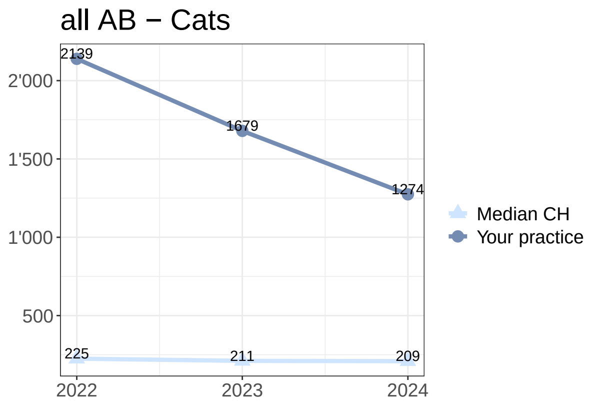

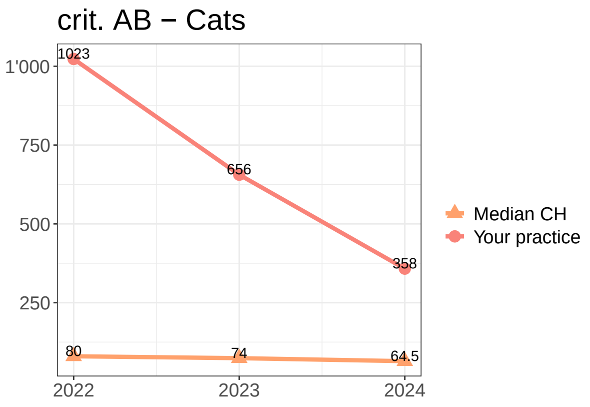

Number of therapy days per animal treated

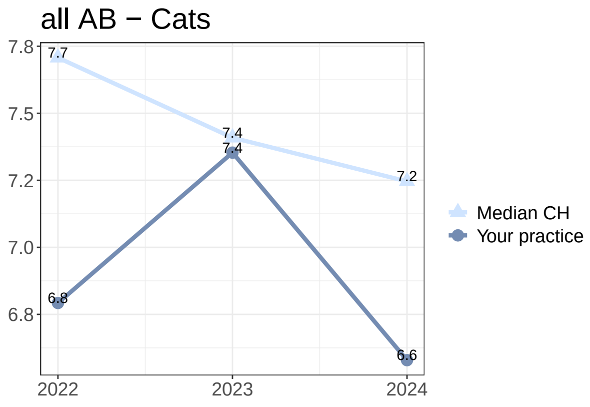

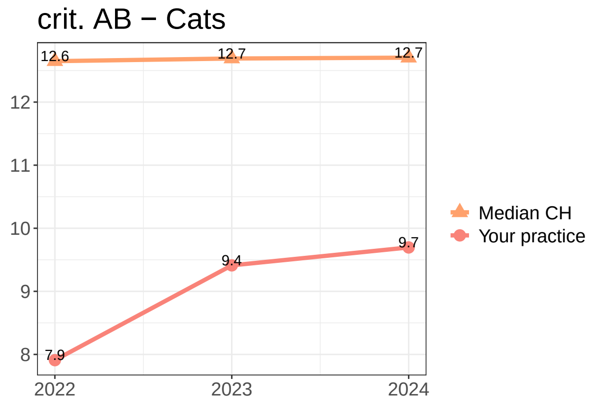

Number of animal treatments per active substance class

Your practice is represented by the blue dot (●); if it does not appear, you have not prescribed any antibiotics of the corresponding class. The other dots represent all practices of a comparable type with more than 100 consultations per year.

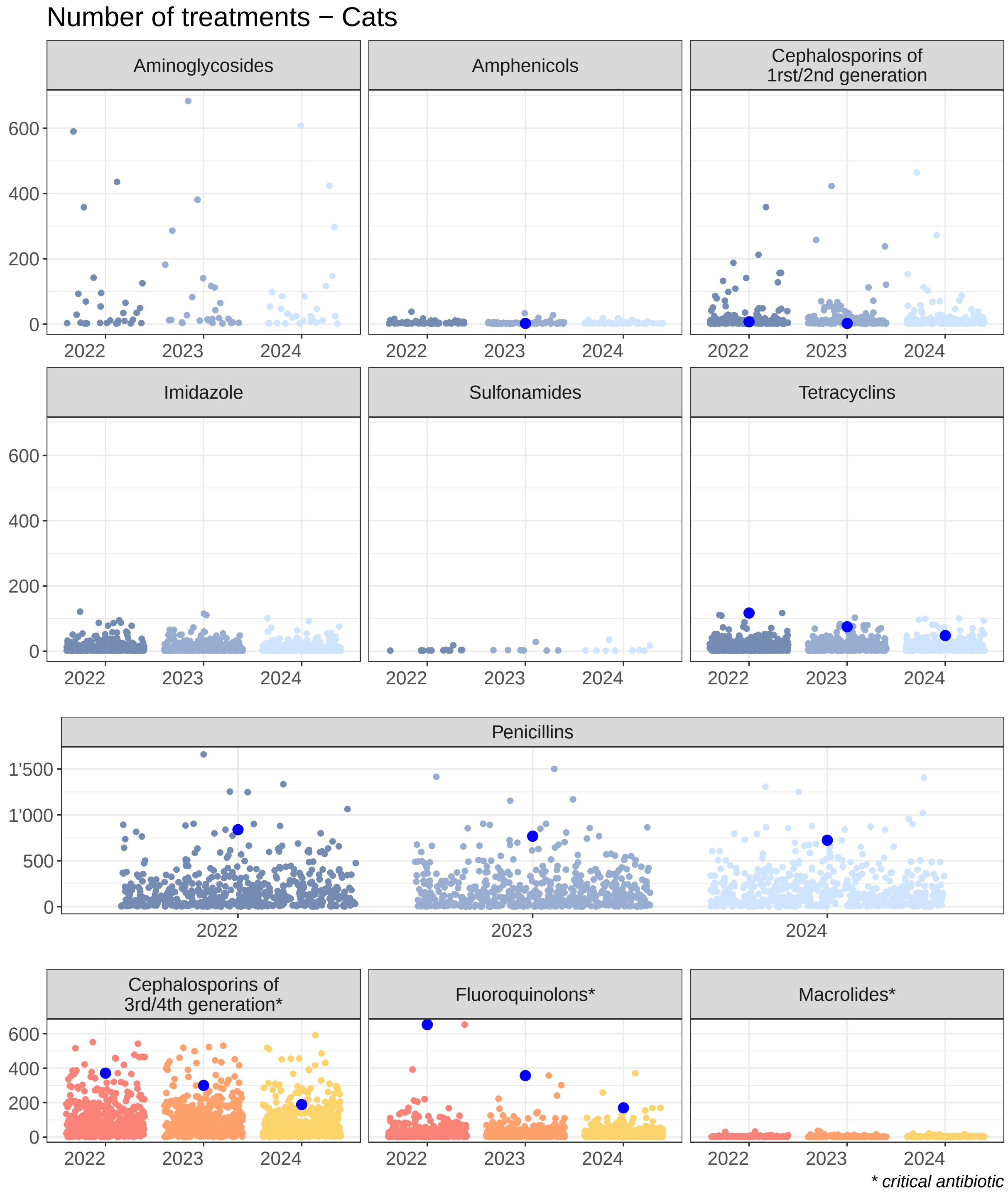

Animal treatments by indication

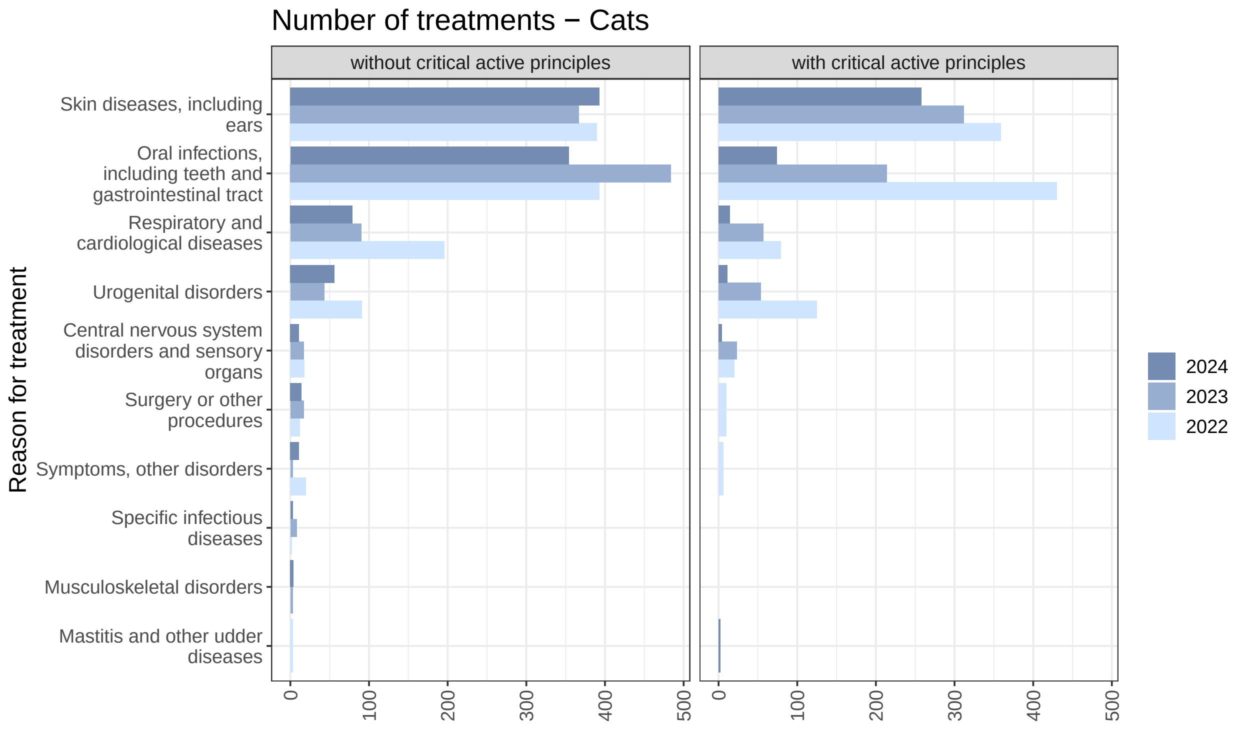

The 10 most frequently used preparations

This chart shows the 10 most frequently used preparations with which you treated the most animals in the last year.

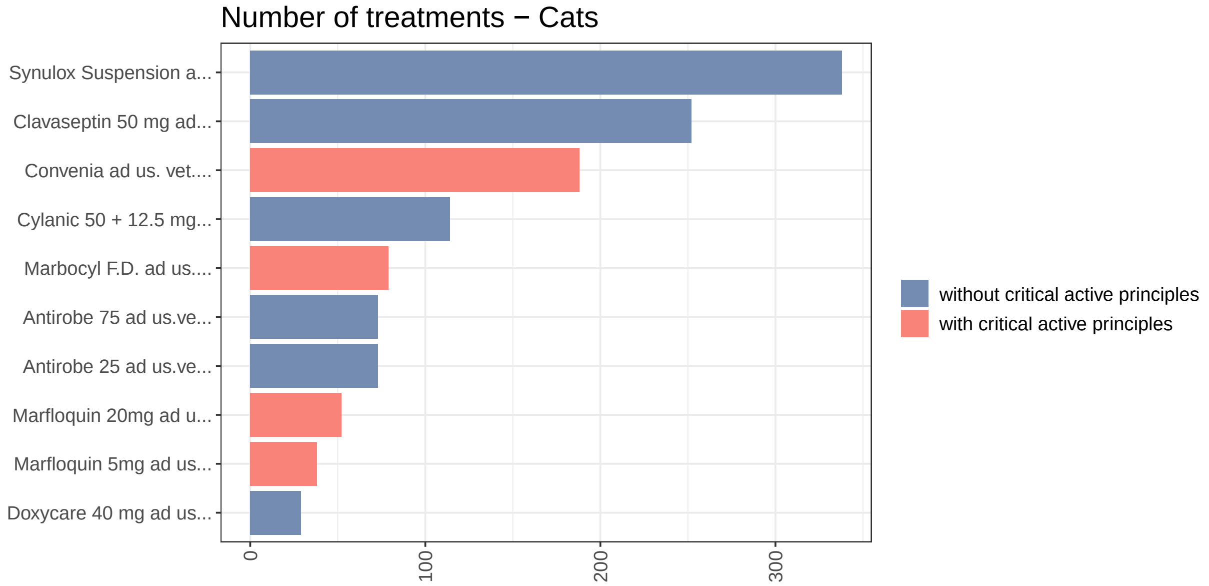

This graph shows all the dosages you prescribed for the preparations listed in the first graph for the year 2024 (amount of active ingredient administered per day and per kg of animal weight).

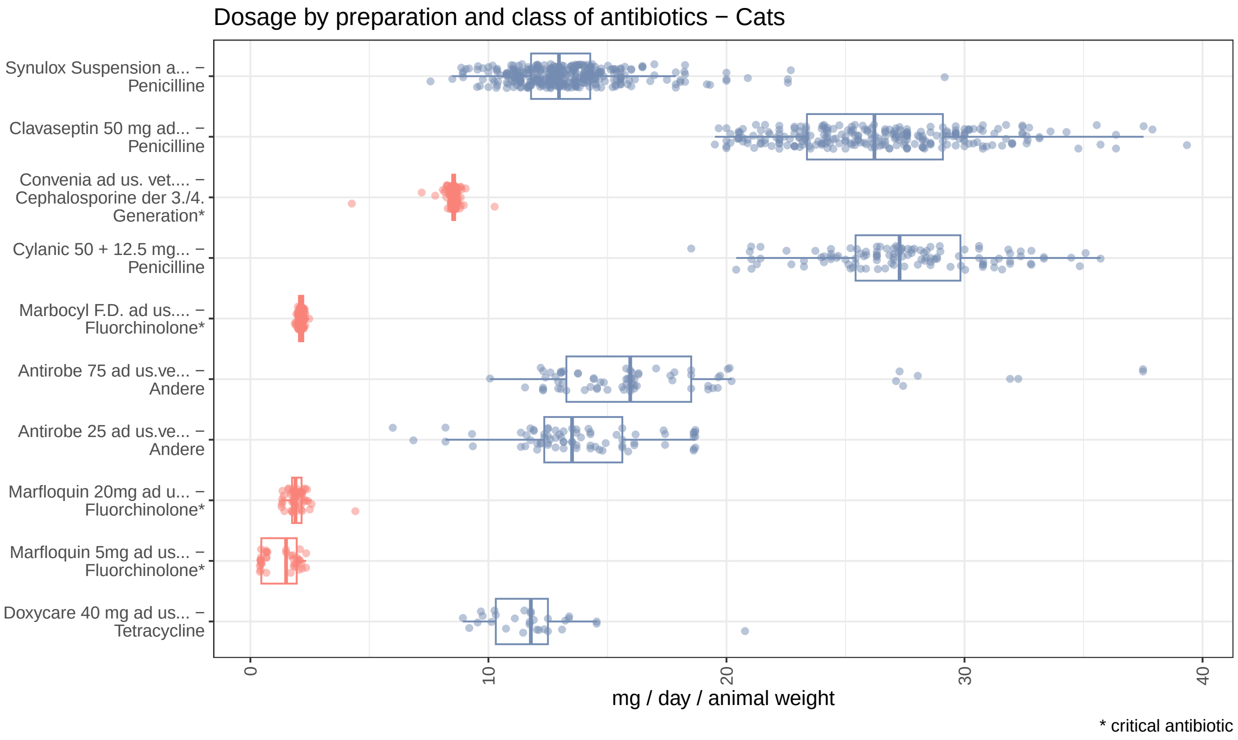

Reading aid

Definitions

- **Consultation**: A consultation is defined as any appointment at a veterinary practice or clinic that leads to treatment or examination by a veterinary surgeon, regardless of whether the animals are treated with antibiotics or other veterinary medicines or not (= 1 entry in the veterinary practice's diary).
- **Prescription**: Meldung einer Antibiotikaverschreibung an das IS ABV.
- **Animal treatment**: AB treatment of an animal.
- **Therapy days**: In the report, therapy days are defined as the sum of the treatment days and the duration of action of the antibiotic. The therapy days prescribed by the veterinary surgeon corresponds to the period between the first and last administration of the antibiotic, supplemented by the action time of the antibiotic. Most preparations have a action time of one day or less. In some preparations, the active ingredients are released over a longer period of time or the active ingredients have a action time of more than one day.
- **Invalid prescription (or unlikely prescription)**: Detailed analyses at the level of individual active ingredients and preparations have shown that individual, presumably incorrect prescriptions can lead to massive deviations in the analyses. Therefore, an exclusion procedure was defined for affected prescription reports: In the first step, all prescriptions were excluded where the dosage calculated from the amount of active ingredient dispensed was more than 15 times the median dosage. In a second step, an outlier detection system was used to remove the most likely anomalies from the data set. The method was developed by the VPHI (Veterinary Public Health Institute, University of Bern) and the FSVO (Federal Food Safety and Veterinary Office). It will be published at a later date and therefore cannot be described in detail here. These prescriptions were excluded from the analysis in this report.

Practice type

The practices were divided into three types. The comparison is made within these types:

- Mixed practice
- Small animal practice with 4,500 or more consultations per year
- Small animal practice with fewer than 4,500 consultations per year
- Equine practice with 1,000 or more consultations per year
- Equine practice with fewer than 1,000 consultations per year

The category may vary depending on the type of animal. The section 'General information' indicates how your practice has been classified.

Evaluation status of practices

All practices receive an evaluation status for the analyses. This categorisation identifies the type of analysis that can be carried out for the respective practice and the corrective measures that have been initiated. There are four groups:

- *‘Number of consultations reported is less than the minimum number for calculating the key figure’*: If there are fewer than 100 consultations per year for an animal species in a practice, only the prescription data is evaluated, but the comparative data is not calculated.
- *‘No consultations reported, therefore no evaluation’*: If no consultations were reported for an animal species in a practice, only the prescription data is evaluated, but the comparative data is not calculated..
- *‘Data incomplete, evaluation possible after processing’*: If the number of consultations was only specified for earlier or later years, this data was used.
- *‘Complete evaluation possible’*

Antibiotic treatment indicator at practice level – pATI

The treatment index for pet practices reflects the intensity of antibiotic treatment in animals. Using the AB treatment indicator (pATI), each practice can compare its antibiotic use for each species of companion animals with the anonymised pATI of other practices. An example of a diagram is shown in the section ‘Example for the presentation of comparative data’.

No pATI is calculated for fewer than 100 consultations per year.

Practices without consultation figures cannot be included in the comparative data process.

Calculation of the pATI

The Animal Treatment Index (pATI) is species-specific and relates the therapy days of antibiotic treatments (numerator) to the number of consultations (denominator).

The **numerator** corresponds to the sum of the treatment days and the duration of action of the active substance for each antibiotic and each animal treated in the year in question. Here are two examples: The prescription of a preparation containing a single active substance with a duration of action of 1 day, administered over a period of 10 treatment days, counts as 10 in the calculation of the treatment duration. A preparation administered over 5 days containing two different antibiotics (each with a duration of action of 1 day) also results in a treatment duration of 10.

The **denominator** is the number of consultations per animal species. It corresponds to the number of animals that had a consultation at the practice and could therefore have received antibiotic treatment. The reason for the visit to the practice is not taken into account. Veterinarians have therefore been obliged since 2023 to send the annual number of consultations for pets to IS ABV. No pATI can be calculated for practices that have not complied with the reporting obligation.

The pATI is calculated per animal species for each practice as follows:

$$\text{pATI} = \frac{\sum \text{for all prescriptions } [(\text{Days of treatment}+\text{Durage of action of the AB}) \text{ for each AB and each animal treated}]}{\text{Nb of consultations}}$$

Signal value and action value

To determine the signal and action values, only practices that have reported their number of consultations and have more than 100 consultations per year are taken into account.

The **signal value** forms the threshold between acceptable and high antibiotic use. It is determined by the 75th percentile, i.e. the pATI value above which 25% of all practices lie. Practices with a pATI above the signal value should check why their antibiotic use is so high and, if necessary, take measures to reduce it as much as possible.

The **action value** forms the threshold between high and very high antibiotic use. It is defined by the 95th percentile, i.e. the pATI of 5% of all practices is above it. Practices with a pATI above the action value should urgently investigate the causes of their very high antibiotic consumption and take action to reduce it as far as possible.

The signal and action values are determined once as described above and then fixed for several years. If AB consumption is reduced, fewer and fewer practices will exceed these values over time than at the beginning.

Species	Type of practice	Action value	Signal value	Action value for crit. AB	Signal value for crit. AB
Dogs	mixed practice	1.57	0.87	0.33	0.13
Dogs	Practice for companion animals < 4500 consultations/year	1.76	0.93	0.43	0.16
Dogs	Practice for companion animals > 4500 consultations/year	1.47	0.88	0.30	0.15
Cats	mixed practice	3.27	1.80	2.19	1.04
Cats	Practice for companion animals < 4500 consultations/year	3.10	1.80	2.27	1.19
Cats	Practice for companion animals > 4500 consultations/year	2.00	1.27	1.50	0.70

The pATI determines the intensity of antibiotic treatments in the practice and the category into which the practice is classified for a particular animal species in comparison with other practices:

- no use of antibiotics (dark green),
- acceptable use of antibiotics (green),
- high use of antibiotics (orange),
- very high use of antibiotics (red).

This report is for information purposes only and does not lead to measures under Art. 36b, c, and d TAMV (Ordinance on Veterinary Medicinal Products, SR 812.212.27). It is intended to give veterinarians an overview of antibiotic consumption in their practice or clinic. It is also intended to help identify whether and where the use of antibiotics could be reduced or optimised.

Practices can take improvement measures on a voluntary basis, for example by consulting the therapy guidelines on the prudent use of antibiotics and educating animal owners about the prudent use of antibiotics: https://www.blv.admin.ch/sachgemaesser-antibiotikaeinsatz.

Example of the presentation of comparative data

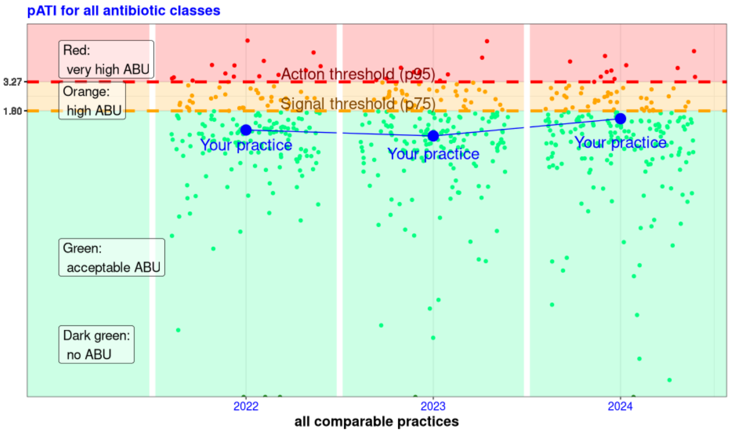

The graphs show the pATI value of your practice (●) compared to the pATI values of all comparable practices. The order of the practices on the X-axis (years) is random and does not represent a ranking. If the blue dot is not present, you have not provided your consultation data for the relevant years. In the example above, the practice has high antibiotic use in 2022 and acceptable use in 2023.

Contact

Please direct any questions or feedback to: isabv@blv.admin.ch
